# Editorial: Self-organizing and excitable signaling networks in cell biology

**DOI:** 10.3389/fcell.2024.1430911

**Published:** 2024-06-04

**Authors:** Chuan-Hsiang Huang, John G. Albeck, Peter N. Devreotes

**Affiliations:** ^1^ Department of Pathology, Johns Hopkins University School of Medicine, Baltimore, CA, United States; ^2^ Department of Molecular and Cellular Biology, University of California, Davis, Davis, CA, United States; ^3^ Department of Cell Biology, Johns Hopkins University School of Medicine, Baltimore, CA, United States

**Keywords:** signaling network, Belousov-Zhabotinsky oscillatory reaction, excitable system, cell migration and proliferation, mathematical modeling, live cell imaging, actin cytoskeletal dynamics

## Introduction

**FIGURE 1:**
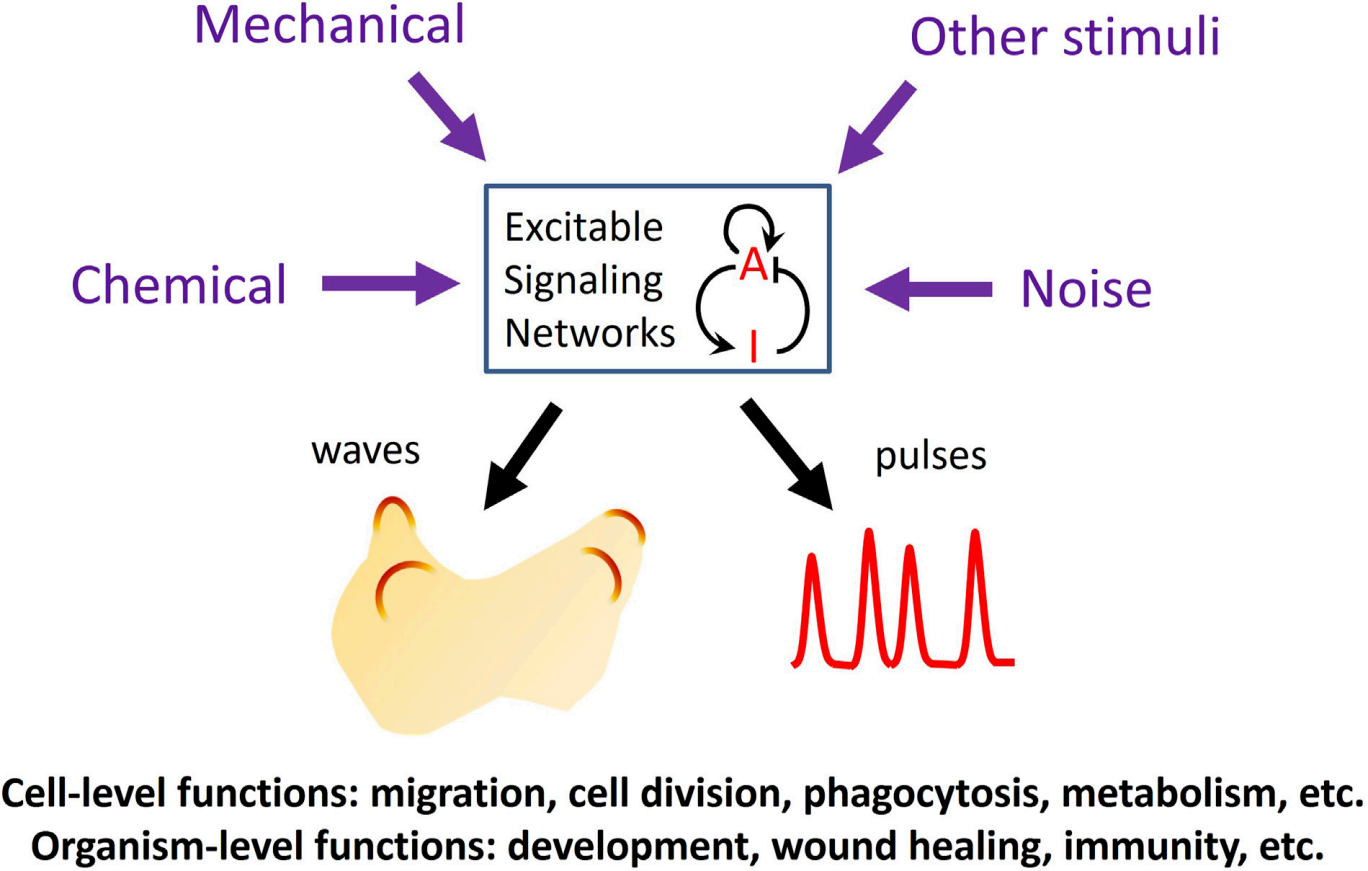
Functions of excitable signaling networks. The activity of an excitable signaling network, represented by a prototypical activator (A)-inhibitor (I) system, can be tuned by various types of external stimuli, which can enter the network through different pathways to modulate the frequency of the waves or pulses of activation. Excitability plays crucial roles in processes at both the cellular and organismal levels.

In the past 2 decades, advances in live cell imaging using fluorescent microscopy have revealed that certain signaling networks display intriguing spatiotemporal patterns characteristic of excitable systems. Classic examples of excitable systems include electrical waves in neuromuscular cells, calcium waves in fertilized eggs, and the Belousov-Zhabotinsky reaction. Excitable systems have been defined in various ways but generally possess the following features: 1) the existence of a resting, an excited, and a refractory state, and 2) the capacity to propagate the excited state as waves. Upon receiving a suprathreshold stimulus, the system transitions from the resting state into an excited state, followed by a refractory, or unresponsive state before returning to the resting state. Waves in excitable systems mutually annihilate due to the system’s inability to reactivate during the refractory period. These properties are also evident in various signaling networks, including the actin cytoskeleton and its regulators and the Ras/PI3K network that operates downstream of numerous receptors. The high temporal resolution of live-cell imaging has revealed that the activities of these networks spontaneously organize into dynamic spatial patterns across a range of scales, spanning from individual cells to populations of cells. While some stimuli trigger excitable activity, others can modulate the threshold for excitation, providing a means to integrate multiple inputs to mediate crucial cellular functions in both prokaryotic and eukaryotic cells (Loose et al., 2011; Huang et al., 2013; Tang et al., 2014; Yang et al., 2018; Di Talia and Vergassola, 2022; Gallagher et al., 2022) (Figure 1).

## Excitability of cortical actin network

Many cellular processes involve changes in cell morphology driven by the polymerization of actin. Live imaging of actin and its regulators revealed that the polymerization activity of the cortical network propagates as mutually annihilating waves (Vicker, 2002; Gerisch et al., 2004; Weiner et al., 2007). These cortical waves have been observed in cells undergoing migration, mitosis, embryonic development, and immune activation. Functionally, cortical excitability coordinates the cellular morphological changes in response to environmental stimuli.

In mast cells, traveling waves and oscillations of F-actin are driven by feedback interactions between signaling molecules such as Cdc42 and FBP17 (Wu et al., 2013). A research article in this issue by Tong et al. provides molecular insights into the nucleation mechanism of these actin waves through antagonism between microtubule and actin polymerization. Specifically, they find that the microtubule-binding formin FHDC1 (FH2 domain-containing protein 1), when released from depolymerizing microtubules, mediates actin polymerization to initiate actin waves. Disruption of FHDC1 causes defective cell polarity and division. These findings add to our understanding of the molecular mechanisms and functions of cortical wave initiation.

Waves of F-actin and Rho activation have also been observed in the eggs and embryonic cells of frogs and starfish. In these cells, cortical waves are regulated by mitotic spindles and are implicated in cytokinesis (Bement et al., 2024). Sepaniac et al. studied how actin waves respond to wounding and change over the course of embryonic development in the *Xenopus* embryo. They found that wounding causes waves to converge and stop, pulling the cell-cell junction toward the wound, possibly contributing to the healing process. Interestingly, over the developmental time course, cortical excitability is replaced by epithelial excitability in which waves of actin and calcium propagate across the cell population rather than individual cell cortices. This observation hints at possible roles of excitability in both individual cells and across the cell population during embryonic development, as has been demonstrated in other processes discussed below.

## From individual cells to cell populations in receptor signaling networks

Ras/PI3K signaling in *Dictyostelium discoideum* has been a particularly revealing model for signaling network excitability operating at multiple scales (Devreotes et al., 2017). Matsuoka et al. review recent progress in *Dictyostelium* cell migration directed by the excitable network involving Ras GTPases and their downstream effectors including PI3K, PTEN, and phosphoinositides PI(3,4,5)P3 and PI(4,5)P2. Within an individual cell, these signaling molecules can undergo spontaneous symmetry breaking to generate polarity and coordinate remodeling of the actin cytoskeleton, driving cell migration. When coupled to upstream gradient-sensing receptors, excitation of the Ras/PI3K signaling network can be spatially biased by chemical gradients to mediate chemotaxis. This excitable behavior of Ras signaling can be simulated mathematically using reaction-diffusion equations.

As a population, *Dictyostelium* cells respond to starvation by initiating a developmental program that culminates in the formation of fruiting bodies. In this process, periodic waves of cAMP, a chemoattractant for *Dictyostelium,* propagate across the cell population to coordinate the collective migration of cells toward each other. Drawing on biochemical and genetic analyses, Jaiswal et al. present an integrated model for the generation of outwardly propagating multicellular waves of cAMP in developing *Dictyostelium*. In their model, multiple positive and negative feedback loops between MEK/ERK and cAMP/PKA signaling pathways underlie the activation, adaptation, and re-activation responses to cAMP. This model underscores the complexity of molecular interactions involved in the generation of oscillatory cAMP waves.

Findings in *Dictyostelium* have led to understanding excitable Ras and actin signaling in higher eukaryotes. For example, waves of Ras-PI3K activation and F-actin can generate protrusions in mammalian epithelial cells, and induce pulsatile activation of ERK, an important regulator of cell proliferation (Albeck et al., 2013; Aoki et al., 2013; Regot et al., 2014; Yang et al., 2018; Goglia et al., 2020; Zhan et al., 2020). Propagation of ERK activity waves across the population can coordinate the collective migration and survival of epithelial cells (Aoki et al., 2017; Hino et al., 2020; 2022; Gagliardi et al., 2021), connecting excitable networks to the physiological function of tissues.

## Excitability in biological vs. chemical systems

Excitable biochemical systems in eukaryotic cells, including the actin and Ras signaling networks, generally involve complex interactions between a large number of molecular species. Such systems are very challenging to analyze in their intact state, due to the difficulty of experimentally manipulating them in the sophisticated ways required to dissect their molecular functions. In contrast, simple excitable systems composed of a handful of components have been found in prokaryotes and chemical reactions. Riedl and Sixt compared the excitable dynamics of polymerizing actin with that of the Belousov-Zhabotinsky reaction, a well studied excitable chemical system composed of five reagents. Despite the vastly different magnitude of their complexities, remarkable similarities are shared between the two systems such as the responses of the propagating waves to geometric constraints or external fields as well as emergent collective behaviors.

## Conclusion

The papers in this Research Topic capture various aspects of the fascinating phenomenon of self-organized and excitable dynamics of signaling networks in different organisms, highlighting their roles in cell migration, embryonic development, and cell proliferation. Since these processes are involved in the pathogenesis of many diseases, future research into the molecular basis and the functions of signaling excitability may lead to new targets for intervention.
